# Poor Sleep Quality Is Associated with Higher Hemoglobin A1c in Pregnant Women: A Pilot Observational Study

**DOI:** 10.3390/ijerph15102287

**Published:** 2018-10-18

**Authors:** Sanika Chirwa, Chioma R. Nwabuisi, Gwinnett M. Ladson, Linda Korley, Janice E. Whitty, Robin Atkinson, John T. Clark

**Affiliations:** 1 Neuroscience and Pharmacology, Meharry Medical College, 1005 Dr. D.B. Todd Jr. Boulevard, Nashville, TN 37208, USA; chioma.nwabuisi@gmail.com (C.R.N.); linda.korley@uphs.upenn.edu (L.K.); 2 Obstetrics and Gynecology, Meharry Medical College, 1005 Dr. D.B. Todd Jr. Boulevard, Nashville, TN 37208, USA; gladson@mmc.edu (G.M.L.); janice.e.whitty@uth.tmc.edu (J.E.W.); robina2010@gmail.com (R.A.); 3 Professional and Medical Education & Internal Medicine, Meharry Medical College, 1005 Dr. D.B. Todd Jr. Boulevard, Nashville, TN 37208, USA; jclark@mmc.edu

**Keywords:** actigraphic wrist-watch, Center for Epidemiologic Studies Depression Scale—Revised, cortisol, impaired glucose tolerance, Sleep Fragmentation Index, Pittsburgh Sleep Quality Index, wake episodes

## Abstract

We hypothesized that poor sleep quality exacerbates glucose intolerance manifested as elevated glycosylated hemoglobin (HbA1c), which increases the risk for gestational diabetes. To test this, 38 pregnant and 22 non-pregnant (age, 18–35 years; body-mass index, 20–35 kg/m^2^) otherwise healthy women were enrolled in the study. Sleep quality was assessed during gestational week 24 (pregnant), or outside of the menstrual period (non-pregnant), using qualitative (Pittsburgh Sleep Quality Index) and objective (actigraphic wrist-watch) measures. Blood glucose, total cortisol, and depression status were evaluated. Eight pregnant and one non-pregnant women were lost to follow-up, or withdrew from the study. There was a higher incidence of poor sleep quality in pregnant (73%) relative to non-pregnant women (43%). Although actigraphic data revealed no differences in actual sleep hours between pregnant and non-pregnant women, the number of wake episodes and sleep fragmentation were higher in pregnant women. Poor sleep quality was positively correlated with higher HbA1c in both pregnant (*r* = 0.46, *n* = 26, *p* = 0.0151) and non-pregnant women (*r* = 0.50, *n* = 19, *p* = 0.0217), reflecting higher average blood glucose concentrations. In contrast, poor sleep was negatively correlated with cortisol responses in pregnant women (*r* = −0.46, *n* = 25, *p* = 0.0167). Three pregnant women had elevated one-hour oral glucose tolerance test results (>153 mg/dL glucose). These same pregnant women exhibited poor sleep quality. These results support the suggestion that poor sleep quality is an important risk factor that is associated with glucose intolerance and attendant health complications in pregnancy.

## 1. Introduction

Pregnancy is a time of significant sleep disturbance, even for women without a history of problems with sleeping [1]. Factors contributing to poor sleep quality include physiological, hormonal, emotional and physical and anatomical changes. However, the relationships between sleep loss during pregnancy and maternal and fetal outcomes are unclear. This was the focus of the present study. Several studies have found that sleep loss in adults causes insulin resistance, elevates evening cortisol, increases sympathetic tone, resulting in impaired glucose tolerance (IGT) [2,3,4,5]. This is of concern because dysregulated glucose metabolism leading to IGT is common in pregnancy, and can be associated with adverse pregnancy outcomes like gestational diabetes (GD) [6,7]. Gestational diabetes is linked to perinatal mortality and morbidity, and GD is increasing in prevalence worldwide [8]. However, the risk factors inducing intolerance to glucose and that leading to GD are unclear [9]. Here, we tested the idea that poor sleep quality exacerbated IGT manifested by elevated glycated hemoglobin (HbA1c), a measure of average blood glucose concentration in the preceding 2–3 months [10]. Thus, we suggest that poor sleep quality during gestation may be an important risk factor triggering IGT and GD in susceptible pregnant women.

During the second half of pregnancy, mothers undergo an “accelerated starvation” (i.e., catabolic state) whereby there is a further increase in insulin resistance. The increased insulin resistance is likely due (at least in part) to the effects of anti-insulin hormones, such as human placental lactogen, progesterone and estrogen [11,12], in addition to “normal” counter-regulatory hormones such as glucagon, cortisol, and catecholamines. Insulin resistance causes a reduction in uptake of dietary carbohydrate, amino acids, and fatty acids by maternal tissue which results in the elevation of postprandial plasma levels of glucose, amino acids and fatty acids to support fetal growth. Most pregnant women adjust well to the hormonal changes and related elevation of maternal plasma glucose, but 4–25% of the antenatal population do not adapt (for reasons that are unclear) and develop glucose intolerance [13,14]. Problematic glucose intolerance (when it occurs) typically appears around week 24 to 28 of gestation, and poses multiple risks to both mother and the growing fetus such as GD and the growth of large babies that are difficult to deliver. Fetuses that are exposed to high glucose levels produce more fetal insulin which accelerates growth (with increased adiposity) termed macrosomia [14]. Consequently, there is a need to identify novel risk factors that predispose women to IGT and its consequences. At present the frequently implicated risk factors (e.g., physical inactivity, obesity, poor diet, and/or smoking) are inadequate for early detection of women at greatest risk for developing glucose intolerance and GD. Surprisingly, little attention has focused on the relationship between sleep quality and impaired glucose tolerance despite the common occurrence of significant sleep disturbances in pregnancy [1,15]. We hypothesized that poor sleep quality exacerbates glucose intolerance (manifested as elevated HbA1c) and, thus, increases the risk for gestational diabetes. However, there is a dearth of evidence-based data on what constitutes adequate sleep (quantitative and/or qualitative) during pregnancy and how this may be associated with IGT and linked to gestational diabetes and other adverse pregnancy outcomes. Furthermore, most studies on sleep in pregnancy lack an appropriate non-pregnant control group. This was addressed in this pilot project whose major goal was to evaluate the relationship between sleep quality and HbA1c in otherwise healthy pregnant and non-pregnant women. A related feature is that glucose intolerance is exacerbated under conditions of physical and psychological stress [16,17]. These factors are potent triggers that causes extra secretion of cortisol, the stress hormone. Cortisol is a strong counter-regulatory hormone, antagonizing the effects of insulin [18]. Excess cortisol could independently exacerbate IGT in pregnancy. Thus, to gain some preliminary insight into the possible effects of cortisol, we evaluated depression levels and total cortisol in relationship with HbA1c. Here, we present data supporting the suggestion that poor sleep quality is a novel risk factor that exacerbates IGT (as reflected in elevated HbA1c) when compared to good sleepers.

## 2. Materials and Methods

### 2.1. Study Participants

We recruited pregnant and non-pregnant women presenting between July 2011 and September 2013 at the Obstetrics and Gynecology (OBGYN) clinic of an academically affiliated hospital in Nashville, TN, USA. Research flyers outlining the study goals and contact information of Study Coordinators were posted within the OBGYN clinic and access areas to the hospital. Participation was voluntary and we targeted both pregnant women during their second trimester and non-pregnant women attending the clinic for routine gynecological care. After obtaining informed consent, each participant was interviewed to obtain demographic information. Race/ethnicity was self-reported (i.e., non-Hispanic Blacks, non-Hispanic Whites, Hispanics, and Other).

There were three study groups namely: (1) Expectant mothers aged between 18–35 years who had not previously borne an offspring, i.e., nulliparous; (2) Expectant mothers in the same age group, who had prior pregnancies that were delivered normally, i.e., multiparous; and (3) Non-pregnant matched controls who had never given birth. The criteria for inclusion were: (1) Healthy women; (2) Singleton pregnancy or non-pregnant; (3) Non-smokers; (4) Not diabetic; (5) Non-asthmatics; (6) No hypertension; and (7) Body-mass index (BMI) between 20 and 35 kg/m^2^. Pregnant women were asked to initially report to the antenatal clinic for examination when they reached 24 weeks of gestation and then go to the Participant and Clinical Interactions Resource (PCIR) center located in the same building wing as the OBGYN clinic. Non-pregnant women were asked to report directly to the PCIR, any day outside of the menstrual period, to undergo basic clinical examination to verify their health status. At the PCIR participants spent 15–20 min completing two study questionnaires and donating blood samples subsequently used to measure HbA1c and total cortisol as described in the following sections. Pregnant women were compensated with up to $100 ($50 for non-pregnant women) to thank them for participation in the study. Participants were free to withdraw from the research study for any reason, at any time. The research study was approved by the Institutional Review Board at Meharry Medical College (Protocol #14-01-122).

### 2.2. Self-Rated Sleep Quality

In order to assess sleep quality participants completed the Pittsburgh Sleep Quality Index (PSQI) developed by Buysse and co-workers [19]. The PSQI is comprised of 19 self-rated questions relating to sleep habits which are grouped into 7 sleep components namely sleep latency, sleep duration, sleep disturbance, subjective sleep quality, habitual sleep efficiency, use of sleep medication, and daytime dysfunction. The PSQI solicited for the most accurate responses on the sleep habits for the majority of days and nights in the preceding 30 days. Subjects scored each component using an ordinal scale between ‘0’ and ‘3’ points whereby ‘0’ indicated no difficulty and ‘3’ indicated severe difficulty. The sum of the component scores yielded a global score from ‘0’ to ‘21’ points where ‘0’ indicated no difficulty and ‘21’ corresponded to severe difficulties in all areas. Cumulative PSQI global scores > 5 reflected poor sleep quality [19]. The PSQI questionnaire is well validated and has very high test-retest reliability and excellent validity for persons with primary insomnia [19,20,21,22]. The PSQI questionnaire has been successfully used in pregnant women e.g., [15,23].

### 2.3. Measurement of Sleep Fragmentation and Duration

Participants were provided an actigraph recorder from Ambulatory Monitoring, Inc (Ardsley, NY, USA), a watch-sized monitor that collects activity and rest periods based on wrist movement and muscle contractions. Actigraph watches contain accelerometer sensors that correctly distinguish sleep from wakefulness approximately 88% of the time relative to standard polysomnographic recordings [24,25]. Furthermore, actigraphic sleep percentage and sleep latency estimates correlate 0.82 and 0.90 with corresponding parameters scored from the polysomnograms, respectively [24]. Participants were asked to wear the watch on their wrist continuously during the day and night to include bedtime sleep hours for seven consecutive days. Each participant went home with instructions to return the actigraphic watch to our staff at the end of the 7-day period. The archived activity data from the sleep watches were downloaded into the ActionW 2.6 sleep scoring software (Ambulatory Monitoring, Inc., Ardsley, NY, USA). We quantified sleep parameters as averaged scores over the seven days. The measurements included night time sleep duration, number of awakenings, sleep efficiency (i.e., time asleep/time in bed), number of naps during the day, and total cumulative time of diurnal sleep episodes [24,26]. Wake episodes reflected the number of blocks of contiguous wake epochs lasting for ≥60 s during bedtime sleep. The sleep fragmentation index was computed from the number of awakenings divided by total sleep time in minutes and multiplied by a hundred [26]. Actigraphic watches are well validated and used by clinicians and researchers to assess sleep hygiene in infants, adults and pregnant women [15,25,27,28,29].

### 2.4. Assessment of Depression Levels

The Center for Epidemiologic Studies Depression Scale—Revised (CESDR) scale was the second questionnaire that was completed by participants. The CESDR scale is comprised of 20 queries grouped in nine categories namely dysphoria, anhedonia, appetite, sleep, thinking/concentration, worthlessness, tiredness/fatigue, agitation/retardation, and suicidal ideation [30]. The CESDR questionnaire required participants to think back to feelings and attitudes in the preceding week and to select the responses that best described how often they felt or behaved that way on an ordinal scale of ‘0’ to ‘4’, from best to worst respectively. To account for response bias, four items are positive mood feelings that are then reverse coded. The scores from each category were added together to yield a CESDR global score with a range between ‘0’ and ‘60’. The CESDR global scores >16 indicated clinical level depression symptoms.

### 2.5. Quantification of HbA1c and Total Cortisol

We collected by venipuncture two blood samples between 9:00 a.m.–11:00 a.m. from each participant while at the PCIR center as follows: the first 5 mL blood sample was collected into heparinized vacutainer tubes and used to assay HbA1c. The HbA1c assesses glycemia reflecting average blood glucose levels in the preceding 1–3 month period [10,31]. The second 5 mL blood sample was used to measure total serum cortisol concentration in blood. In both cases, the specimens were picked up by Quest Diagnostics (Atlanta, GA, USA) on the same day who subsequently conducted the diagnostic clinical assays. Quest Diagnostics performs clinical HbA1c assays by immunoturbidimetry methods, and quantification of total cortisol is done through liquid chromatography/tandem mass spectrometry.

### 2.6. Oral Glucose Tolerance Test (OGTT) and Ponderal Index

The timing for the collection of the above blood samples coincided with the timing when each pregnant woman was screened for oral glucose tolerance test by her healthcare provider, respectively. Briefly, during the 24-week gestation phase, most of the pregnant women met with their health care providers and were made to orally consume 50 g of a glucose solution as part of a routine antenatal screening. Subsequently, blood samples were taken after 60 min to test for blood glucose levels, respectively. GD was queried if the 1-h post-50 g OGTT score was ≥153 mg/dL [32,33]. We requested pregnant women to submit copies of their OGTT results to the Study Coordinators where available. Lastly, after delivery of the baby, we obtained information on type of delivery, weight and height of the neonate from the medical records. Some of the data were used to calculate the ponderal index, a measure of adiposity of the infant using the following equation: PI = (baby weight in gram × 100)/(crown-to-heel length in cm)^3^ [34,35,36,37].

### 2.7. Statistical Analyses

The data were analyzed using GraphPad Prism 6 for Windows (GraphPad Software, San Diego, CA, USA. Between-group comparisons of continuous variables were analyzed with one of the following: *t*-tests and/or one-way ANOVA with Tukey’s post-hoc test. Data fitness for normal distribution was verified with D’Agostino and Pearson Omnibus normality test [38]. Pearson product moment correlation was used to determine associations between sleep quality and HbA1c or total cortisol. Tests were two-tailed with *α* = 0.05. A sample size of 14 had an 80% power to detect differences of 2.08 in the average PSQI global scores between pregnant and non-pregnant women (*α* = 0.05, two-tailed).

## 3. Results

### 3.1. Profiles of Study Cohort

Thirty-eight pregnant women (31 Black or African-American, four White, one Native American, one Asian, and one more than one race) met the criteria for inclusion and participated in the study. Pregnant women consisted of 16 nulliparous and 22 multiparous women. In addition, data were collected from 22 non-pregnant women (21 Black or African American, one Asian) who had never given birth. There were insignificant differences in age (years: non-pregnant, 24.7 ± 0.6; nulliparous, 24.00 ± 1.5; and multiparous, 26.9 ± 1.3; F(2, 47) = 1.818, *p* = 0.1735), and body-mass index (kg/m^2^: non-pregnant, 25.5 ± 0.9; nulliparous, 27.2 ± 0.9; and multiparous, 28.2 ± 1.1; F(2, 46) = 1.889, *p* = 0.1628) among the three groups. Values are reported as mean ± SEM in this and subsequent entries. Out of the 38 pregnant women, six were subsequently lost to follow-up, one was withdrawn and referred to appropriate medical care because of severe clinical depression. Two women (one pregnant and one non-pregnant) were excluded from further data analyses for taking prescription sleep medication. This left a total of 30 pregnant (14 nulliparous and 16 multiparous) and 21 non-pregnant women who were included in the final data analyses. Furthermore, the small numbers of Hispanics and non-Hispanic Whites precluded stratification based on race/ethnicity. Thus, the study participants were predominantly non-Hispanic Blacks.

### 3.2. Pregnancy Changes Sleep Quality

**Figure 1:**
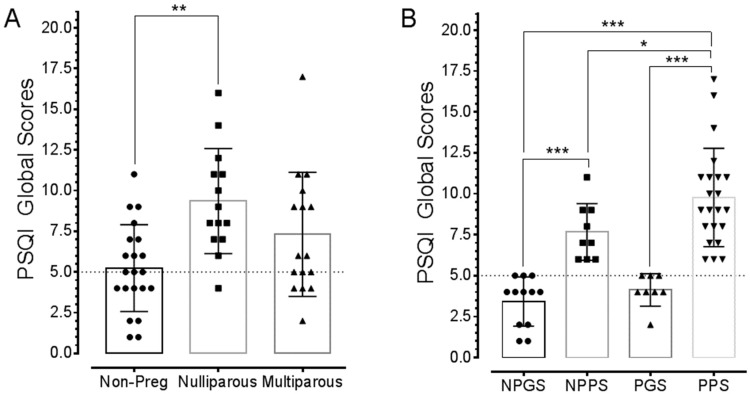
Sleep quality in pregnant and non-pregnant women. (**A**) Data obtained from the PSQI questionnaire showing sleep quality in pregnant and non-pregnant women. PSQI global scores > 5 (dotted line) indicate poor sleep quality with severity corresponding to higher scores. Average PSQI global scores were highest in nulliparous women. (**B**) Data shows that pregnant poor sleepers (PPS) presented with more severe poor sleep quality compared to both non-pregnant good sleepers (NPGS) and pregnant good sleepers (PGS). Note that sleep quality was worse in PPS relative to non-pregnant poor sleepers (NPPS). Asterisks indicate *p* values for One-way ANOVA with Tukey’s post-hoc as follows: * *p* < 0.05, ** *p* < 0.01, *** *p* < 0.001.

The PSQI results revealed a higher incidence of poor sleep quality in pregnant than non-pregnant women. Thirteen (92.8%) of the nulliparous and 9 (56.3%) of the multiparous women exhibited poor sleep quality (i.e., PSQI global scores >5). By contrast, only 9 (42.9%) of the non-pregnant women presented with poor sleep quality. PSQI global scores were higher in the nulliparous group compared to both multiparous and non-pregnant women (F(2, 48) = 7.015; *p* = 0021; Data in Figure 1A).

In terms of reported sleep hours, these were insignificant differences among the groups (bedtime sleep hours: non-pregnant, 6.86 ± 0.19; nulliparous, 6.29 ± 0.57; multiparous, 7.50 ± 0.48; F(2, 36) = 1.884, *p* = 0.1667). Data fitness for normal distribution was confirmed with D’Agostino and Pearson Omnibus normality test (K2 values: non-pregnant, 4.026; nulliparous, 7.495; multiparous, 5.284; *p* > 0.05). Subsequently, participants were assessed in sub-groups based upon their sleep quality, i.e., good sleepers (i.e., PSQI global scores ≤5) or poor sleepers (i.e., PSQI global scores >5) and status (pregnant or non-pregnant, Figure 1B). Pregnant women were grouped together because of the low number of good sleepers among nulliparous women (Figure 1A).

### 3.3. Pregnant Poor Sleepers Report Difficulties Falling Asleep at Bedtime

**Figure 2:**
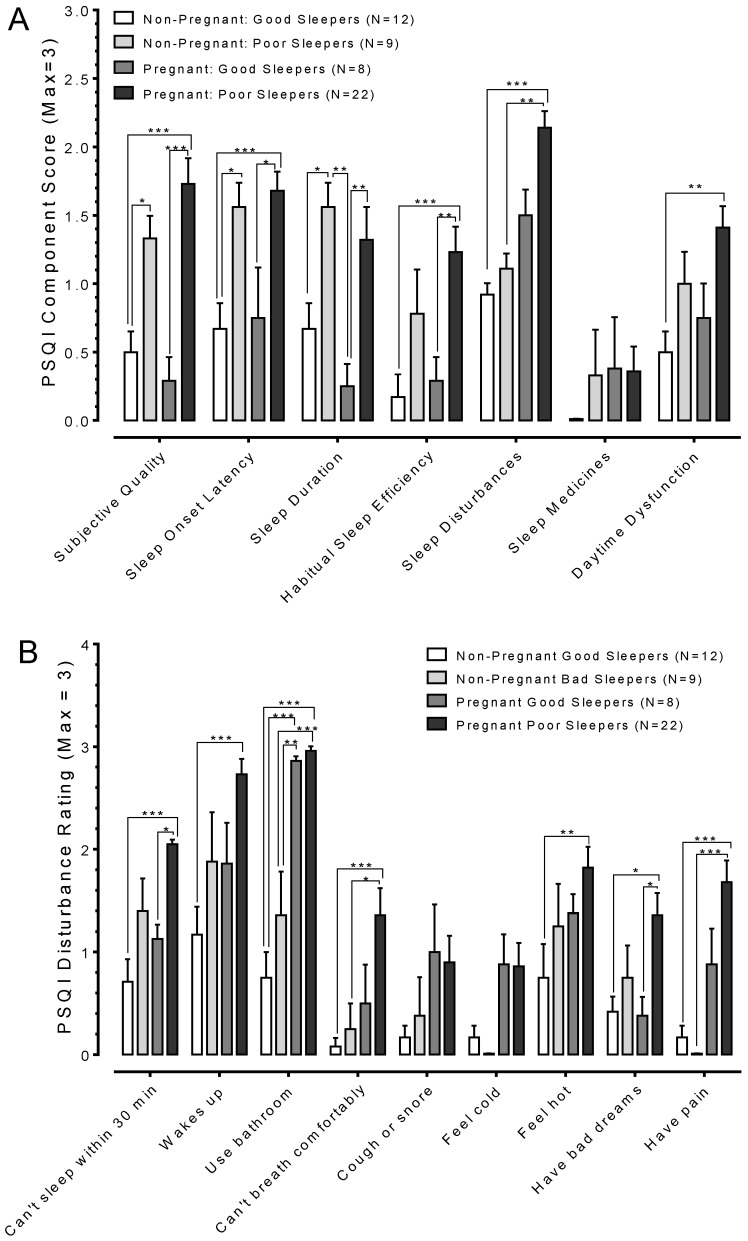
PSQI component and sleep disturbances scores. (**A**) Plots of aggregate responses to the seven components that make up the PSQI questionnaire. Pregnant poor sleepers recorded the worst scores in the majority of the sleep components relative to non-pregnant good sleepers. (**B**) Further analysis of factors contributing towards the sleep disturbance component reveals that use of the bathroom and waking up during bedtime sleep were the major types of disturbances. Asterisks indicate *p* values for One-way ANOVA with Tukey’s post-hoc as follows: * *p* < 0.05, ** *p* < 0.01, *** *p* < 0.001.

Analysis of the seven sleep components of the PSQI was conducted to gain insights on the factors contributing towards poor sleep quality. The majority of poor sleepers (pregnant or non-pregnant) rated their sleep as being ‘fairly bad’, and this was particularly true for pregnant poor sleepers (data in Figure 2A). Poor sleepers reported relatively longer periods before falling asleep once in bed, with asleep onset latency typically ranging between 30 and 60 min, whereas it was less than 30 min for good sleepers (pregnant or non-pregnant). Furthermore, poor sleepers felt they slept fewer hours out of the total number of hours spent in bed relative to good sleepers.

Only pregnant poor sleepers reported significant daytime dysfunctions, i.e., difficulties with staying awake while driving, eating meals, or engaging in social activity. Pregnant women reported more serious forms of sleep disturbances in comparison to non-pregnant women, regardless of their sleep quality (data in Figure 2B). In addition to delayed sleep onset, pregnant poor sleepers reported waking up “in the middle of the night or early morning” more frequently (i.e., three or more times a week) than did non-pregnant women (i.e., less than once a week). During bedtime sleep, pregnant poor sleepers reported having difficulties with breathing, felt hot, experienced bad dreams, and/or felt pain relative to non-pregnant good sleepers (data in Figure 2B).

### 3.4. Sleep is Greatly Fragmented in Pregnant Poor Sleepers

**Figure 3:**
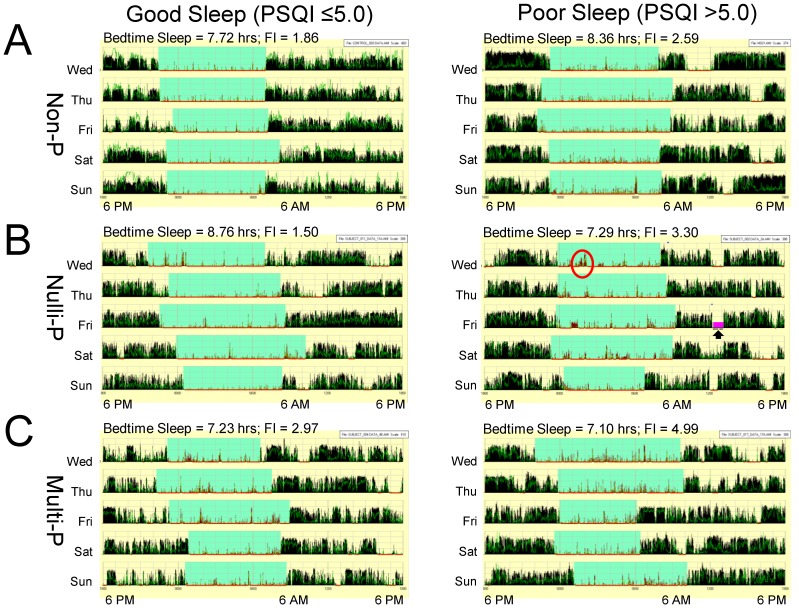
Representative linear actigrams recorded from six women in research study. Each row of the linear actigram is a full 24-h daily cycle starting at 6 PM and the light green shaded regions indicate bedtime sleep, respectively. In (**A**) are data from a non-pregnant participant (Non-P), (**B**) reflects a nulliparous subject (Nulli-P), and actigrams shown in (**C**) are from a multiparous pregnant woman (Multi-P). Actigraphic watches could detect when wrist-watch was not worn (black arrow) and/or bad recording segments (purple shading). Red circle is an example of a long wake episode during bedtime sleep. Notice the lower sleep fragmentation index (FI) shown on top of each actigram for good sleepers relative to women with poor sleepers in the three research study groups, respectively.

**Figure 4:**
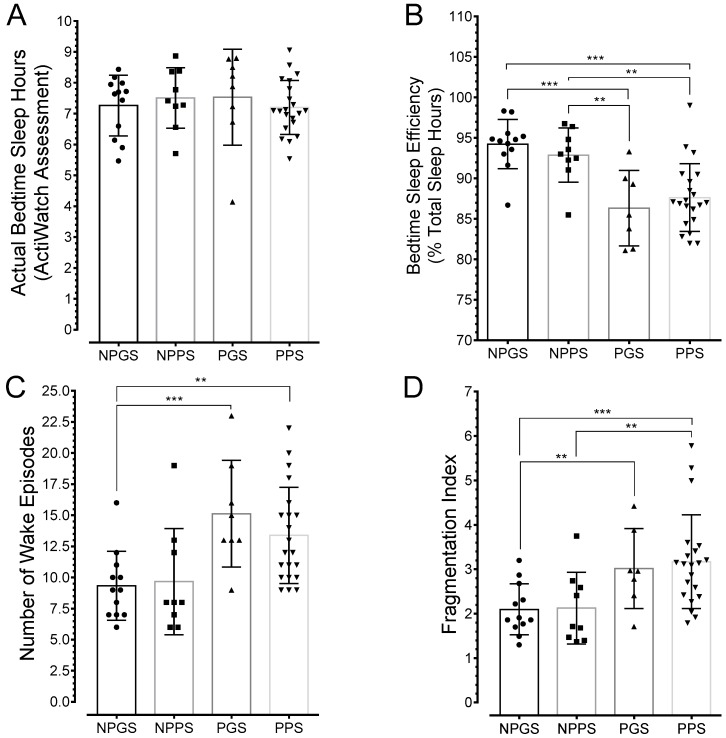
Averaged activity and sleep data from actigraph recordings. (**A**) Actual bedtime sleep hours were the same among the groups. (**B**) Plots reveal significant reductions in sleep efficiency in pregnant women relative to non-pregnant women. (**C**) The average number of wake episodes were significantly higher in pregnant women compared to non-pregnant good sleepers. (**D**) Similarly, sleep fragmentation was significantly greater in pregnant women relative to non-pregnant good sleepers. Asterisks indicate *p* values for one-way ANOVA with Tukey’s post-hoc as follows: * *p* < 0.05, ** *p* < 0.01, *** *p* < 0.001.

A measure of actual sleep duration was obtained from linear actigrams retrieved from actigraph watches worn on the arm wrist continuously for 7 days. Representative linear actigrams are shown in Figure 3 and data are presented in Figure 4.

There were no differences in actual bedtime sleep duration between pregnant and non-pregnant women (F(3, 46) = 0.3103, *p* = 0.8178; Figure 4A). This confirmed the findings obtained with the PSQI scores. Interestingly, there were no differences (F(3, 46) = 0.3383, *p* = 0.7977) in total sleep amount in a circadian cycle (i.e., includes bedtime sleep and daytime naps) between pregnant and non-pregnant women regardless of sleep quality (% total sleep content in a 24-h period: non-pregnant good sleepers, 33.75 ± 1.91, *n* = 12; non-pregnant poor sleepers, 35.17 ± 2.04, *n* = 9; pregnant good sleepers, 36.05 ± 2.65, *n* = 8; and pregnant poor sleepers, 35.52 ± 0.87, *n* = 21). However, differences were found in bedtime sleep efficiency, i.e., actual sleep hours divided by time in bed, (F(3, 45) = 5.286, *p* = 0.0033); pregnant women exhibited relatively poor bedtime sleep efficiency (Data in Figure 4B). In addition, the average number of wake episodes were higher in pregnant women compared to non-pregnant good sleepers (F(3, 45) = 11.17, *p* < 0.001; Data in Figure 4C). Similarly, the sleep fragmentation index was higher in pregnant women relative to non-pregnant good sleepers (F(3, 46) = 5.879, *p* = 0.0017; Figure 4D). Fragmentation index reflected the number of awakenings divided by total sleep time in minutes and multiplied by 100 [26].

### 3.5. Poor Sleep Quality is Associated with Depression

**Figure 5:**
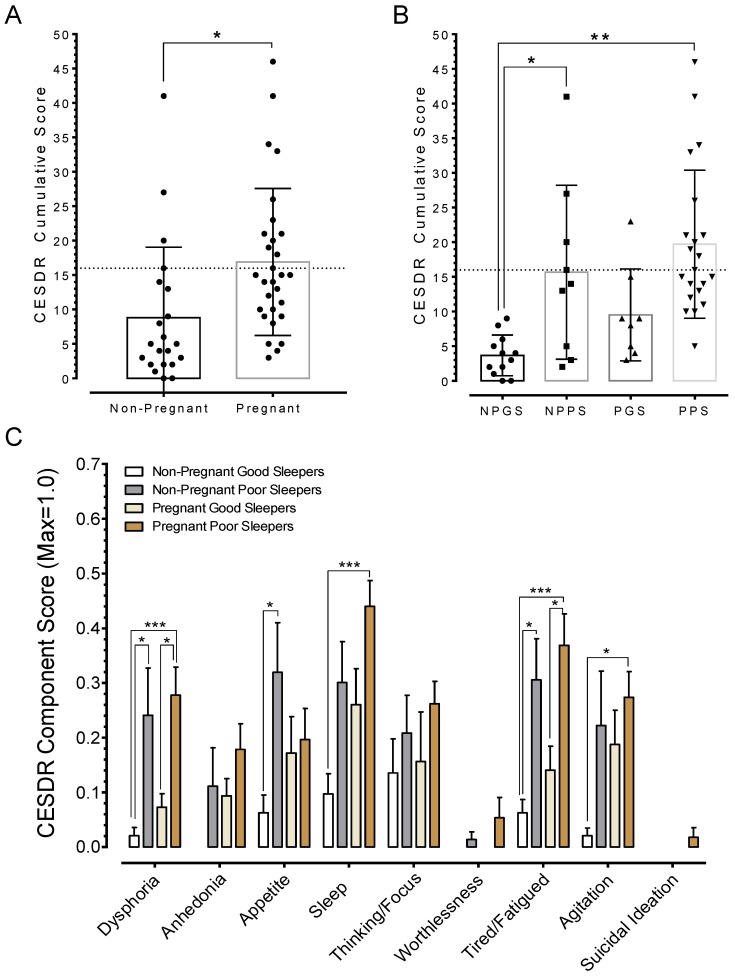
Relationship between sleep quality and depression. (**A**) Aggregate CESDR global scores for pregnant and non-pregnant women. (**B**) A division into subgroups revealed that poor sleepers had higher CESDR scores than good sleepers in both groups, but only poor sleepers among pregnant women showed clinical level depression symptoms. (**C**) The plot shows the nine self-rated categories making up the CESDR questionnaire. *Y*-axis reflects normalized ratings up to a maximum score that can be obtained in each category. Notice that dysphoria, sleep, and fatigue ranked higher among the factors affecting the ratings. Asterisks indicate *p* values for One-way ANOVA with Tukey’s post-hoc as follows: * *p* < 0.05, ** *p* < 0.01.

Next, the association between sleep quality and depression level was evaluated. Thus, data collected using the Center for Epidemiologic Studies Depression Scale–Revised (CESDR) scale was analyzed. It should be noted that CESDR global scores range between 0 and 80 and values > 16 indicate clinical level depression symptoms [30]. The results showed significant differences, t(48) = 2.689, *p* = 0.0098 (data in Figure 5A), in CESDR global scores between pregnant and non-pregnant women. Specifically, poor sleepers in both pregnant and non-pregnant women exhibited higher CESDR global scores relative to non-pregnant women (data in Figure 5B). An evaluation of the individual components making up the CESDR scale revealed significant differences particularly in the sleep items as follows: pregnant poor sleepers had trouble getting to sleep, reported restless sleep, and/or indicated they slept much more than usual relative to non-pregnant good sleepers (data in Figure 5C). In general, poor sleepers were prone to exhibit dysphoria and fatigue when compared to good sleepers. The results also indicated that pregnant poor sleepers were more agitated than non-pregnant good sleepers.

### 3.6. Poor Sleepers Exhibited Elevated HbA1c and Lowered Total Cortisol Levels than Good Sleepers

**Figure 6:**
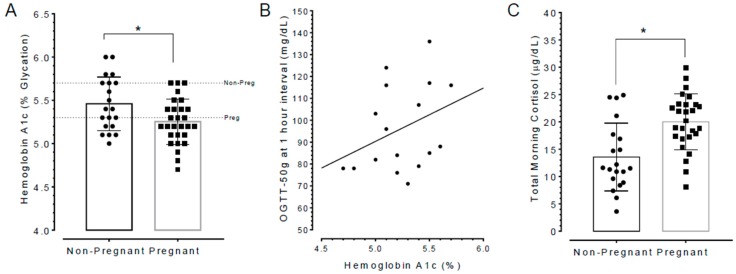
Hemoglobin A1c, OGTT, and cortisol assays. (**A**) Group data showing HbA1c scores from participants in the research study. Cut-off HbA1c in pregnant women in the population with high risk for gestational diabetes like African Americans is 5.3 (lower dotted line), whereas it is 5.7 for non-pregnant women (upper dotted line). (**B**) Oral glucose tolerance test (OGTT) scores showed a positive correlation with HbA1c scores. (**C**) Total cortisol concentrations in pregnant women were higher relative to non-pregnant women but this was still considered within normal ranges for pregnant women [39,40]. Asterisks in figure indicates *p* < 0.05 for unpaired t-test, two-tailed.

Percent hemoglobin A1c, as well as cortisol concentrations in blood samples from participants were measured. It was found that the mean HbA1c was lower in pregnant women relative to non-pregnant women (t(46) = 2.494, *p* = 0.0163; data in Figure 6A). It should be noted that the cut-off HbA1c level in pregnant women in the population with high risk for GD (e.g., African Americans) is 5.3, whereas it is 5.7 for pre-diabetic status in non-pregnant women [32]. By these criteria, 10 (35.7%) of pregnant women exceeded the recommended HbA1c limit, but only 4 (20%) of non-pregnant were above their limits, respectively (Figure 6A). The relatively higher number of pregnant women presenting with HbA1c values that exceeded recommended levels raised the prospect that this could affect OGTT outcomes and this was evaluated. Most of the pregnant women took the 50 g oral glucose tolerance test conducted by their antenatal care physician during gestation week-24 of gestation. Seventeen out of the 30 pregnant women subsequently submitted copies of the OGTT results to the Study Coordinators. The results showed a positive correlation between HbA1c and OGTT scores at the 1-h test interval (*r* = 0.35, *n* = 17, *p* = 0.1746; Figure 6B). One pregnant poor sleeper presented with OGTT scores at the 1-h interval that was suggestive of ‘pre-diabetic’ states (i.e., >135 mg/dL) but review of the patient’s record did not have follow-up screens to confirm gestational diabetes.

In terms of total cortisol concentrations (reflective of the 9:00 a.m. to 11:00 a.m. collection time interval) these were found to be higher in pregnant women relative to non-pregnant women (t(45) = 3.894, *p* = 0.003; data in Figure 6C). It needs emphasizing that during pregnancy cortisol levels increase and attain concentrations that are 2–3 times higher than in non-pregnant states [39,40]. Thus, total cortisol levels recorded in our study were within the normal ranges for healthy pregnant women.

**Figure 7:**
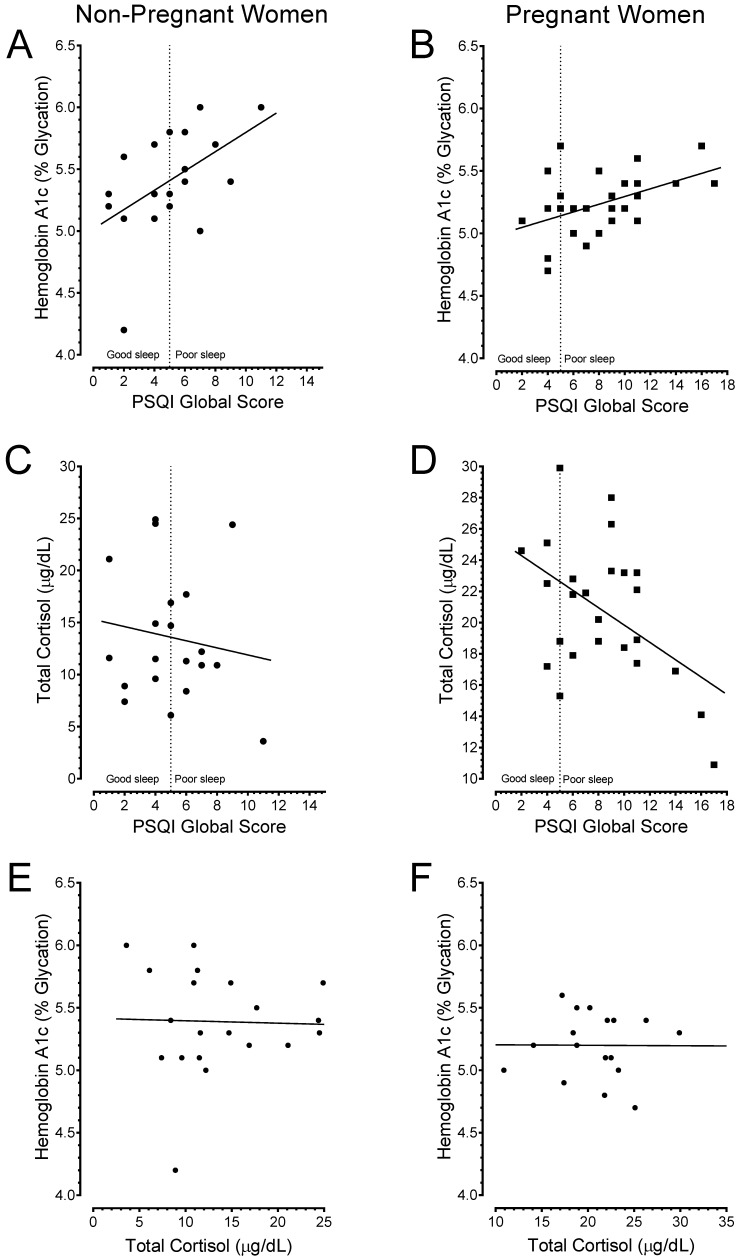
Poor sleep quality is correlated with elevated HbA1c and lower cortisol. Top row shows HbA1c in non-pregnant (**A**) and pregnant women (**B**). The Pearson product-moment correlation coefficient revealed a positive correlation between poor sleep quality and HbA1c in both non-pregnant women, *r* = 0.50, *n* = 19, *p* = 0.0217, and pregnant women, *r* = 0.46, *n* = 26, *p* = 0.0151. In bottom row, the results reveal that the cortisol for non-pregnant (**C**) and pregnant women (**D**), respectively. Poor sleep quality corresponded with low cortisol concentrations in non-pregnant (*r* = −0.14, *n* = 20, *p* = 0.5536) and was significant in pregnant women (*r* = −0.46, *n* = 25, *p* = 0.0167). Total cortisol concentrations lacked correlations with HbA1c in both on-pregnant (**E**) and pregnant women.

Next, a Pearson product-moment correlation coefficient was computed to assess the relationship between sleep qualities versus glucose or cortisol concentrations. Data are shown in Figure 7. There was a positive correlation between poor sleep quality and HbA1c in both non-pregnant women (*r* = 0.50, *n* = 19, *p* = 0.0217; data in Figure 7A, B) and pregnant women (*r* = 0.46, *n* = 26, *p* = 0.0151). By contrast, there was a negative correlation between poor sleep quality and total blood cortisol levels in pregnant women (*r* = −0.46, *n* = 25, *p* = 0.0167; data in Figure 7C,D). No correlation was observed between sleep quality and total blood cortisol in non-pregnant women (*r* = −0.14, *n* = 20, *p* = 0.5536). Interestingly, no correlation was found between total cortisol levels and HbA1c scores (data in Figure 7D,E). This suggested that the increased levels of cortisol could not account for the elevation in HbA1c observed in poor sleepers.

### 3.7. Poor Sleep Quality Is Associated with a Higher Incidence of Adverse Pregnancy Outcomes

Finally, the antenatal records were reviewed to determine parturition outcomes and it was found that 29 were full-term deliveries and 1 was a preterm cesarean delivery. Twenty-eight of the 29 full term births were vaginal deliveries but 1 was by cesarean delivery. In addition, baby’s birth height and weight were extracted from the medical files where information was available and used to calculate the ponderal index (PI), a measure of baby’s adiposity or ‘fatness’. It was found that three babies had PI > 3.0 which was suggestive of large babies [34,35,36,37]. Interestingly, these relatively large babies were born to pregnant women that presented with poor sleep quality.

## 4. Discussion

We have presented data demonstrating that otherwise healthy pregnant women had a higher incidence of poor sleep quality than non-pregnant women. The severity of the sleep deficit was greatest in nulliparous pregnant women. We have also shown that there are no differences in actual sleep hours as measured qualitatively with PSQI and objectively with actigraphic watches between otherwise healthy pregnant and non-pregnant women. In contrast, we found differences in the number of wake episodes during bedtime sleep, and the incidence of severe sleep fragmentation was higher in pregnant than non-pregnant women. More importantly, we discovered positive correlations between poor sleep quality and elevated HbA1c in both pregnant and non-pregnant women. By contrast, cortisol levels did not correlate with any changes in HbA1c values. Taken together, these findings render support to the idea that poor sleep quality is a novel risk factor that exacerbates glucose intolerance manifested as elevated HbA1c. The prospects are that poor sleep quality is an antecedent risk factor of adverse pregnancy outcomes like gestational diabetes. This is further discussed in detail below.

Our study was robust because we combined qualitative and objective measures of sleep quality and duration. Specifically, the PSQI questionnaire is well validated in the field and it has high test-retest reliability and a good validity for persons with primary insomnia [19,20,21,22]; it has been successfully used in pregnant women [23]. Similarly, the actigraphic watches are well validated and used by clinicians and researchers to assess sleep hygiene in infants, adults and pregnant women [25,27,28,41,42,43,44]. Thus, we can unequivocally state that self-reported poor sleep quality corresponded with increased number of both wake episodes and severity of sleep fragmentation. Our study is also significant for revealing these findings in non-Hispanic Black women because this population has a high risk for GD [14,41]. Sleep fragmentation is a hallmark of sleep-maintenance insomnia, which differs from sleep onset insomnia [45,46]. Individuals can generally get to sleep relatively quickly after going to bed. However, fragmented sleep means bedtime sleep cycles invariably do not include much slow-wave sleep and is relatively unrefreshing and this negatively impacts hormonal rhythms and metabolism [47,48]. For example, sleep loss causes insulin resistance, elevates evening cortisol, increase sympathetic tone, resulting in impaired glucose tolerance [5]. Consequently, it is tempting to conclude that the elevated HbA1c recorded in poor sleepers was due to the exacerbation of the underlying IGT the third-trimester of pregnancy [48] caused by insulin resistance [49].

An alternative explanation to account for the elevated HbA1c documented in our study could be ascribed to the effects of the counter-regulatory hormone cortisol. However, our preliminary results were at variance with the involvement of cortisol in fostering the elevation of HbA1c. We discovered that good sleepers exhibited both higher total cortisol concentrations and lower HbA1c values relative to poor sleepers. These results were counter-intuitive with expectations but a plausible explanation may lie in the following. Pregnant women begin to feel the strain of their expanding abdomen, particularly during the third trimester, as the uterus is now very close to the rib cage. We speculate that the attendant physical and psychological disturbances invariably trigger coping responses orchestrated by the release of corticotrophin-releasing hormone (CRH) from the paraventricular nucleus of the hypothalamus [50]. Subsequently, CRH stimulates both the synthesis and secretion of adrenocorticotropic hormone (ACTH) from the anterior pituitary gland. ACTH release, in turn, causes the secretion of cortisol from the adrenal cortex and cortisol mediates the amelioration of stress. Thus, the more cortisol released within normal ranges the stronger is the handling of the stress. Not surprising total cortisol concentrations begin to increase during the second trimester [39] and attain levels that are 2–3 times higher than in non-pregnant states [40]. In addition to stress and pituitary ACTH, the mother’s steep rise in circulating cortisol is further affected by CRH secreted by the placenta [51]. Ordinarily, ACTH is secreted in a diurnal rhythm with peak levels in early morning hours and lowest concentrations at night in healthy adults [39,52,53]. Levels also rise independently of circadian rhythm in response to stress and this gives rise to pulsatile cortisol release profiles during the day [54]. However, hypothalamic secretion of CRH also acts as a transmitter in areas of the brain that are responsible for the neuropsychological responses to stress [50]. Thus, a robust cortisol response reflects higher CRH activities that also acts to ameliorate the physical and/or psychological stresses. The more robust this response is, within normal ranges, the stronger would be the attendant stress relief. This may have accounted for the improvement in sleep quality observed good sleepers and also presented with higher cortisol levels relative to poor sleepers. Taken together, our data render support to the proposition that that poor sleep quality, but not rise in cortisol levels, is a risk factor that exacerbates IGT as manifested by elevated HbA1c values. The combination of glucose intolerance secondary to poor sleep quality, coupled with a predisposition to a “diabetic state” that occurs during the second-half of pregnancy [11,12], may tilt the balance towards pregnancy complications like gestational diabetes. This requires further investigation.

## 5. Conclusions

To conclude, our study draws attention to the need for regular assessment of both sleep quality and HbA1c as part of routine antenatal care. Monitoring and evaluating good sleep quality as well as HbA1c can help guide non-drug interventions for curtailing adverse pregnancy outcomes like GD [55]. Recently published reports add credence to this idea [56,57,58,59]. Our study was limited in scope in that we evaluated HbA1c during the third trimester only. The study did not directly assess insulin resistance per se to verify its linkage to poor sleep quality. Similarly, we only evaluated spot cortisol concentrations without controlling for factors such as food intake. To assess the level of stress it is necessary to carry out multiple measurements of blood cortisol during the day. Our study cohort was predominantly composed of non-Hispanic Black women. Clearly larger studies involving multiple racial/ethnic groups are needed to verify if regular assessments of both sleep quality and HbA1c, during the three trimesters, will identify pregnant women at greatest risk for GD.
